# Psychological Group-Treatments of Social Anxiety Disorder: A Meta-Analysis

**DOI:** 10.1371/journal.pone.0079034

**Published:** 2013-11-15

**Authors:** Hanna Wersebe, Marit Sijbrandij, Pim Cuijpers

**Affiliations:** 1 Department of Clinical Psychology, VU University, Amsterdam, The Netherlands; 2 EMGO Institute for Health and Care Research, Amsterdam, The Netherlands; 3 Leuphana University Lüneburg, Lueneburg, Germany; University of Melbourne, Australia

## Abstract

**Background:**

A few meta-analyses have examined psychological treatments for a social anxiety disorder (SAD). This is the first meta-analysis that examines the effects of cognitive behavioural group therapies (CBGT) for SAD compared to control on symptoms of anxiety.

**Method:**

After a systematic literature search in PubMed, Cochrane, PsychINFO and Embase was conducted; eleven studies were identified that met the inclusion criteria. The studies had to be randomized controlled studies in which individuals with a diagnosed SAD were treated with cognitive-behavioural group therapy (CBGT) and compared with a control group. The overall quality of the studies was moderate.

**Results:**

The pooled effect size indicated that the difference between intervention and control conditions was 0.53 (96% CI: 0.33–0.73), in favour of the intervention. This corresponds to a NNT 3.24. Heterogeneity was low to moderately high in all analyses. There was some indication of publication bias.

**Conclusions:**

It was found that psychological group-treatments CBGT are more effective than control conditions in patients with SAD. Since heterogeneity between studies was high, more research comparing group psychotherapies for SAD to control is needed.

## Background

Both population surveys [1] and studies [2]; [3]; [4] indicate that Social Anxiety Disorder (SAD) is a highly prevalent psychiatric disorder. The National Comorbidity Survey-Replication [1] for example, provides prevalence estimates of 12-month and lifetime DSM-IV SAD of 6.8% and 12.1%, respectively. SAD is defined by a marked fear of social performance situations and to possible scrutiny by other people [5]. As a result, phobic situations may be avoided or only endured with intense anxiety, and a pronounced distress and impairment in daily occupational and social life results. A diagnosis of SAD has been found to be associated to scholastic difficulties, as these individuals are more likely not to finish high school and to fail a grade [6]. Accordingly, SAD is related to reduced quality of life [7], impaired functioning and high (economic) costs [8]. However, SAD is still one of the least well-recognized mental disorders [e.g. 9] and frequently under-diagnosed [10].

To decrease the burden of patients various treatments have been developed. One of the most broadly researched and applied treatments for SAD is cognitive-behavioural therapy (CBT). Cognitive-behavioural therapies typically include a vast range of techniques, such as exposure to social stimuli and tasks and cognitive restructuring [11]. During exposure, the client is exposed to feared (social) situations despite experiencing distress. Cognitive restructuring is applied to correct maladaptive beliefs about the self and others. In addition, social skills training and applied relaxation are commonly used in treatment of SAD. Further, all techniques involve repeated practice both in form of homework and in the therapeutic setting.

Various past studies have supported the efficacy of cognitive-behavioural group therapy (CBGT) in diverse populations [12]; [13]; [14]; [15]; [16]; [17]. In CBGT, the therapy is carried out in a group setting. Group treatments, where patients with the same disorder are treated at the same time, may have certain benefits since social situations can be simulated more easily, mutual support might be given and exposure to social situations may occur naturally. Also, group therapies may be more cost-effective than individual therapies since they take less therapist time per patient [18].

Until now, only a few meta-analyses have examined the effects of psychological treatments for SAD. Specifically, five meta-analyses have been conducted in the last two decades that exclusively examined effects of psychotherapy on SAD [19]; [20]; [21]; [22]; [23]. Most of them were hampered by methodological limitations since, for example, only a few high-quality randomized controlled trials (RCTs) were available at the time they were conducted [20]; [21]; [22]; [23]. That could have resulted in an inflated effect size and therefore in a distorted overall picture. Although all previous meta-analyses report controlled effect sizes, which are known to be more conservative, only the meta-analysis of Acarturk et al. [19] applied tests for heterogeneity of included primary studies. Those state-of-the-art analyses are crucial in order to find differences between studies and their specific impact on the outcome of interest. Moreover, they can give an indication of treatment effects in specific population.

Unlike the earlier meta-analyses [20]; [21]; [22]; [23], Acarturk et al. [19] included only RCTs on the available psychological treatments for SAD. Earlier findings of the abovementioned meta-analyses could be replicated with the asset of detailed analyses of subgroups. Furthermore, all meta-analyses on SAD have combined both studies on individual and group treatment formats and no study until now had explicitly focused on CBGT contrasted to a control group only. Group-treatments and their particular effect-sizes are crucial to investigate as the limited resources of health care providers and their constant cutbacks on expenses for psychotherapies engage interest in both the most economical and effective treatment at the given point of time.

Therefore, the aim of our study was to investigate the effectiveness of CBGT based on the most recent published trials compared to waiting-list, placebo or treatment-as-usual (TAU) conditions in adults with SAD on reducing social anxiety symptoms. Hence, it is expected that CBGT is more effective in reducing social anxiety symptoms than control.

## Method

### Study Eligibility

Studies were included when (1) the effects of group-treatments (2) in subjects aged 18 years or older (3) with a principal diagnosis of SAD (4) were compared with a control condition (5) in an RCT.

Moreover, studies had to define SAD either according to the Diagnostic and Statistical Manual of Mental Disorders (DSM-III-R [24] or DSM-IV [5]) or contain a fixed cut-off score on clinician-rated SAD questionnaire (Table 1). Control conditions could be treatment-as-usual (TAU), pill-placebo or waiting list. Studies were excluded, when the standardized mean difference could not be calculated (usually the case when no conducted statistical test examined the difference between the psychotherapy and the control condition).

**Table 1:** Basic characteristics of the studies.

First author	Country	Age groupyears	Recruit-ment	Diagnosis	Type of SAD	Conditions	Sub-jects(n)	Intervention (Number of sessions)	Follow- up	Instrument	DO (%)	ITT/CO
Blanco (2010)	USA	18-61	Clin + com	SM-IV (SP primary)		CBGT/placebo	39/40	CBGT: automatic thoughts, logical errors, behavioural tasks, exposure, CR (12)	Pre, post	LSAS, ADIS, CGI-S, FQ, SIAS, SPS, CGI-I, SDS	27.87	ITT
Davidson (2004)	USA	18-65	com	DSM-IV: GSP	GSP	Comprehensive CBT/Placebo	60/59	Comprehensive CBT: in vivo exposure, CR, SST (14)	Pre, post	BSPS, CGI-S, SPAI	28.0	ITT
Gruber (2001)	USA	25-60	com	ADIS-R: SP (according to DSM III-R)		CBGT/WL	14/17	CBGT: exposure, CR, generalization/maintenance (12)	Pre, Post, 6-months	FNE, SPAI, SPS	14,8	Co
Heimberg (1998)	USA	18-65	Clin + com	DSM-III-R SP		CBGT/placebo	28/27	CBGT: automatic thought, logical errors, formulation of rational responses, assignments for exposure, self- administered CR (12)	Pre, post	SAD, FNE, FQ, LSAS, SAD, SCL-90-R anxiety, SIAS, social avoidance SPS	22,5	CO
Hofmann, (2004)	USA	18 +	Clin	DSM-III-R		CBGT/WL	26/19	CBGT: skills to identify negative cognitions, in vivo exposure, homework (12)	Pre, post, 6 months	SPAI	22.5	CO
Hope (1995)		18-59	clin	DSM- III -R SP		CBGT/WL	13/10	CBGT: combined CR; roleplayed exposure, homework (12)		SADS, FAH, FNE	18	CO
Mörtberg (2006)	EU	< 65	com	DSM- III-R SCID	GSP/non-GSP	ICBGT/WL	13/13	ICBGT: psychoeducation, CR, AR, homework, video-recorded exposure	Pre, post, 3 and 6 months	LSAS, SPS, SIAS	4.55	CO
Mörtberg (2007)	EU	18-65	Com	DSM-IV SP		ICBGT/TAU	26/18	CBGT: skills to identify negative cognitions, with exposure, homework (16)	Pre, post, 8 and 12 months	SIAS, SPS, LSAS, FNE, FQ, SPWSS, SBQ,	28.0	ITT
Rapee (2007)	AU	20-65	Clin +com	DSM-IV	GSP	group treatment/ WL	59/52	Group treatment: CR, exposure, feedback, attention training, home exercises (10)	Pre, post, 3 months	SIAS, SPS, BFNE	37,5	ITT
Stangier (2003)	EU	18- 65	Clin+ Com	DSM-IV SP (SCID)		ICBGT/WL	22/21	CBGT: CR, exposure, behaviour experiments, Clark & Wells model	Pre, post, 3 and 6 months	SPAI, SPS, SIAS	8.5	CO
Wong (2006)	China	18-60	Clin	DSM- IV		CBGT/WL	17/17	CBGT: psycho-education, identify negative cognitions, developing strategies, exposure, identifying and dealing with core beliefs (10)	Pre, post	LSAS	/	CO

CBGT, Cognitive-behavioral group therapy; WL, Waiting List; LSAS, Liebowitz Social Anxiety Scale; CGI-S, Clinical Global Impressions Scale; FQ, Fear Questionnaire; SIAS, Social Interaction Anxiety Scale; ADIS, Anxiety Disorders Inventory Schedule; ADIS-R, Anxiety Disorders Inventory Schedule–Revised; SIAS, Social Interaction Anxiety Scale; SPS, Social Phobia Scale; CGI-I, BSPS, Brief Social Phobia Scale; FNE, Fear of negative evaluation; Social Interaction Anxiety Scale; SPWSS, Social phobia weekly Summary Scale; SBQ, Social Behavior Questionnaire; ADIS-R, Anxiety Disorders Inventory Schedule–Revised; SPAI, Social Phobia and Anxiety Inventory – social phobia ; SDS, Sheehan Disability Scale; SAD, Social Anxiety Disorder; SADS, Social Avoidance and Distress Scale; DO, Drop-Out; Co, Completers; ITT, Intention-to-Treat; CBGT, cognitive-behavioral group-treatment; CBT, Cognitive-behavioral Therapy; DSM, Diagnostic and Statistical Manual of Mental Disorders; CR, Cognitive Restructuring; IR, interpersonal relationship

### Search Strategy

Studies were retrieved through systematic literature searches (from 1980 to 22 March 2012) in the databases of PubMed, Cochrane Central Register of Controlled Trials, PsychInfo and embase. Searches were conducted by means of databased keywords and text words indicative of SAD, while limiting the search to effect studies (randomized trials, controlled trials, clinical trials). No language restrictions were applied. The complete search strategy is available in the additional file A. Search Strings and results in the Appendix S1.

First, inclusion criteria were applied to titles and abstracts. Potential eligible studies were retrieved in full text. Second, criteria were applied to full articles. Two independent reviewers carried out the data extraction. Disagreement was solved in discussion and consensus.

### Quality assessment

The validity and quality of the studies was assessed with the Cochrane risk of bias tool [25]. This tool allows to determinate the following four possible sources of bias in RCTs: 1. Adequacy of sequence allocation; 2. Allocation concealment; 3. Blinding of assessors and outcomes; 4. Incomplete outcome data (intention-to-treat analyses); as well as selective reporting and other biases. Two independent reviewers assessed the quality of the studies and disagreement was solved by discussion.

### Meta-Analysis

For each comparison between a psychological treatment and a control group, the effect size indicating the difference between the two groups at post-test (Hedges' g) and the 95% Confidence Intervals (CI's) were calculated. Hedges' g was chosen as the present meta-analysis includes several studies with a small sample size and this effect size adjusts for such small sample sizes. Effect sizes were calculated by subtracting (at post-test) the average score of the psychological treatment group from the average score of the comparison group, and dividing the result by the pooled standard deviations of the two groups. An effect size of 0.5 thus indicates that the mean of the experimental group is half a standard deviation larger than the mean of the control group. Effect sizes of 0–0.41 can be assumed to be small, of 0.40 to 0.70 moderate and 0.70 and above as a large effect [25].

Only those scales and measurements that explicitly measured symptoms of anxiety were used in the calculation of the effect sizes. If more than one measure was used to assess the change of SAD, the pooled effect size was calculated, so that each study only provided one effect size. When means and standard deviations were not reported, p-values were used to calculate effect sizes.

The Comprehensive Meta-Analyses computer program (CMA; version 2.2.021) was used to calculate the pooled effect size. Since considerable heterogeneity among the studies was expected, the pooled effect sizes were calculated with the random effects model. In the random effects model it is assumed that the included studies are drawn from ‘populations’ of studies that differ from each other systematically (heterogeneity). In this model, the effect sizes resulting from included studies not only differ because of random error within studies (as in the fixed effects model), but also due to true variation in effect size from one study to the next. Statistical effect sizes are generally not easy to comprehend and interpret from a clinical point of view. For this reason, the effect sizes were transformed into the numbers-needed-to-be-treated (NNT) by using the formulae provided by Kraemer & Kupfer [26]. The NNT indicates the number of patients that have to be treated in order to yield one additional positive outcome in one of them [27].

To assess heterogeneity between studies the I^2^ statistic was calculated, with a value of 0% indicating no heterogeneity, 25% low, 50% as moderate and 75% high heterogeneity [28].

Also, the Q- statistic was calculated to assess the level of homogeneity. A significant p-value indicates that the null-hypothesis of homogeneity has to be rejected and it is likely that the heterogeneity is not due to sample error.

Subgroup analyses were conducted according to the mixed effects model. In this model, studies within subgroups are pooled with the random effects model, which uses random effects within subgroups and fixed effects between subgroups.

Publication bias was tested by inspecting the funnel plot, and by means of Duval and Tweedie's trim and fill procedure [29], which gives an estimate of the effect size after the publication bias has been taken into account. Further, the Begg & Mazumdar rank correlation test [30] was applied to test whether the adjusted and observed effect sizes differed significantly from each other.

## Results

**Figure 1:**
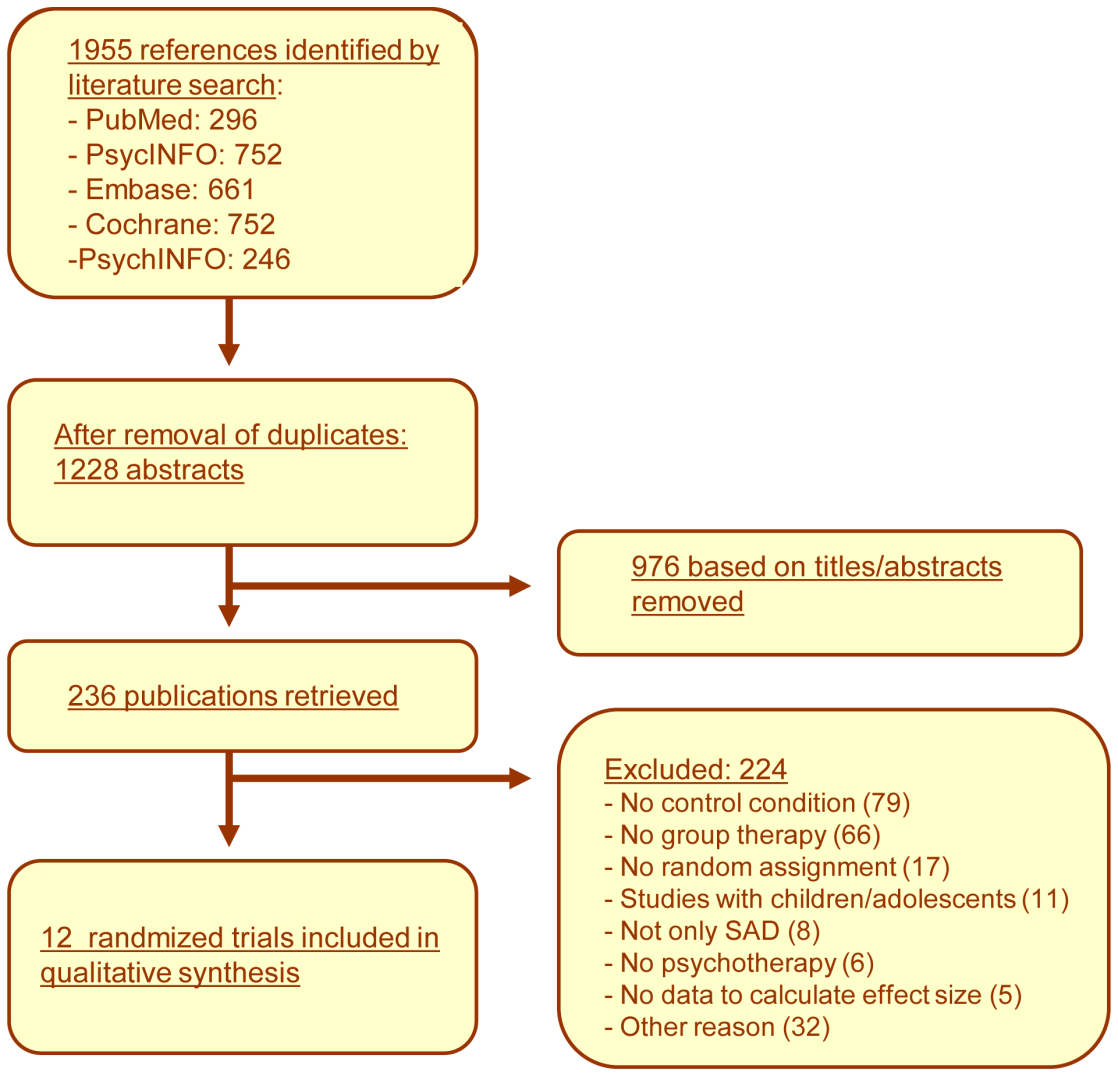
Flowchart of selection and Inclusion of Studies.

In Figure 1, a flowchart describing the inclusion of studies, is presented. Having examined a total of 1955 abstracts (1228 after removal of duplicates), 236 full-text papers that possibly met the inclusion criteria were examined for further consideration.

Two studies were excluded, as no data for the control conditions to which the subjects were randomized were given [15]; [16]. Further, one study had to be excluded since no data were provided for meta-analysis [31].

### Characteristics of included studies

The eleven primary studies [32]–[41] included a total of 654 participants (315 in the treatment conditions and 290 in the control conditions). Selected characteristics of the eleven included studies are described in Table 1. In four studies, subjects were recruited from both community and clinical settings, in four studies from community settings only, whereas in three other studies participants were recruited from clinical settings only. In seven studies psychological treatment was compared with a waiting-list control group, in three studies with a pill-placebo and in one study with TAU.

CBGT was defined as a psychological intervention, in which the following elements were included: 6–16 sessions of therapy led by 2 therapists, 2 or 2.5 hours each and group sizes of at least 4 participants. Moreover, both exposure in vivo and cognitive elements such as cognitive restructuring or skills to identify negative thoughts were part of the treatment.

### Quality Assessment

The quality of the eleven studies varied. In five studies [32]; [33]; [38]; [39]; [40] the allocation to conditions was reported adequately, whereas in the other studies it was not [11]; [34]; [35]; [36]; [37]; [41]. Only two studies described concealment of allocation [32]; [33]. Incomplete data was addressed in most studies [11]; [32]; [33]; [36]; [38]; [39]; [40]; [41]. Percentages of patients lost to follow-up ranged from zero to 37.5%. Intention-to-treat analyses were conducted in only four studies [32]; [33]; [38]; [39].

### Effects of psychological treatments at post-tests

**Figure 2:**
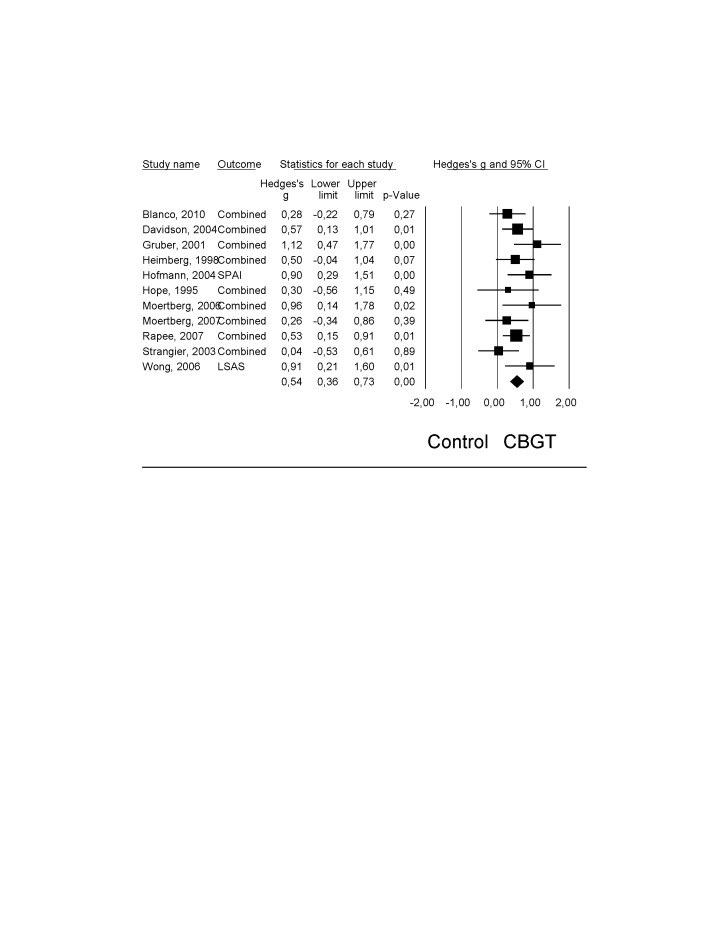
Effect sizes of CBGT for adult SAD: Hedges’ g.

The effects of psychological group-treatment could be compared to a waiting-list, placebo or TAU in eleven studies (Table 2). The mean effect size was g = 0.54 (95% CI; 0.36–0.73), that corresponds with a NNT of 3.36. Heterogeneity was moderate (Q = 11.61; p = 0.31; I^2^ = 13,90%). Figure 2 presents the effect sizes and 95% confidence intervals of each study.

**Table 2:** Meta-analyses of studies examining the effects of group-therapy on social phobia.

		N	Hedges'g	95% CI	Q	I ^2^(%)	P^a)^	P^b)^	NNT
Overall effects									
All studies		11	0.54	0.36–0.73	11.61	13. 90	0.31		3.36
FNE		5	0.55	0.26–0.83	4.15	3.62	0.39		3.31
LSAS		5	0.52	0.21–0.82	3.87	0.00	3.87		3.50
Subgroup analyses									
Control group	Waiting List Placebo+ TAU	74	0.640.42	0.39–0.880.14–0.70	9.171.08	34.550.00	0.160.78	0.25	2.864.27
Recruitment	Community Clinical+ community	38	0.620.52	0.26–0.980.29–0.75	3.637.96	44.928.98	0.160.36	0.64	2.963.50
Analyses	ITT Completers only	56	0.450.68	0.22–0.680.39–0.97	1.298.78	0.0045.48	0.860.12	0.23	4.002.70

a) This p-value in this column indicates whether the Q statistic was significant or not.

b) This p-value indicates whether the subgroups differ significantly

### Subgroup analyses

Since the number of included studies was small, only the most basic subgroup analyses containing at least 4 studies were conducted. Specifically, these were analyses comparing the type of control group (waiting-list *versus* placebo/treatment as usual); type of recruitment (community sample *versus* both community and clinical sample) and type of analyses (intention-to-treat *versus* completers only) (Table 2). There were no statistical subgroup differences with respect to control groups, type of recruitment or type of analyses.

Heterogeneity varied between the subgroups. With the exception of two subgroups, placebo and treatment-as-usual (*I^2^* = 0.00, g = 0.42) and intention-to-treat (*I^2^* = 0.00, g = 0.45), all subgroup analyses resulted in *I^2^* levels higher than 30%. Because of the small number of studies in each subgroup, the results of these analyses have to be interpreted with caution.

### Publication Bias

The funnel plot and Duval & Tweedie's trim and fill procedure indicated some publication bias. After adjustment for missing studies, the effect size dropped from g = 0.54 to g = 0.48 (95% CI: 0.28–0.67; number of trimmed studies: 2) and the Begg & Mazumdar rank correlation test did not indicate a significant difference (p = 0.27, one-tailed).

## Discussion

This meta-analysis found that cognitive- behavioural group-treatments have a moderate, but significant effect in the treatment of SAD compared to control. Some individual studies did not have enough power to detect a significant effect, but after pooling of the studies the effect size became significant. However, the effect sizes differed highly between studies, ranging from zero (indicating no treatment effect) to above one (indicating a large effect size).

With the exception of Acarturk et al [19] the previous meta-analyses [20]; [21]; [22]; [23] included only few RCTs and did not apply tests for heterogeneity and other meta-analytic analyses since they may not have been available. As indicated, those state-of art analyses allow finding differences between studies and their influence on the outcome. Beyond that, none of the earlier meta-analyses investigated the effects of psychological group-formats, specifically CBGT, in SAD in adults. In short, this present meta-analysis extends the prior work by focussing explicitly on CBGT and including only recent RCTs, analysed with the latest available methodology.

This study has nevertheless several limitations. First, the number of included studies was rather small, which may have limited the possibilities to examine potential moderators of outcome. Second, there was a considerable difference between the included studies that may have contributed to heterogeneity. For instance, only few studies conducted intention-to-treat analyses, which are crucial to get a more reliable estimate of the effect size. Thirdly, a meta-analysis is always limited by the methodological quality of included studies. Since we also included studies with lower quality, it may have influenced our overall outcome.

More research in the field of group treatments for SAD is certainly needed. Future studies should give specific attention to important quality criteria, such as adequate randomization procedures and reporting, concealment of allocation, and the inclusion of all randomized respondents in the final analyses. Further, future trials should contain larger sample sizes, and may compare CBGT to other cost-effective therapies. A small study in 37 SAD patients suggested that individual CBT delivered through the internet was equally effective as face-to-face CBGT [42], but associated with substantially less clinician time.

Despite the limitations of this meta-analysis, evidence shows that CBGT seems to be an effective way of treating individuals with SAD. Thus, CBGT may be an attractive alternative to individual face-to-face CBT to decrease the burden of SAD, because of its advantages with respect to cost-effectiveness and since vivo exposure to social situations may occur naturally during group sessions.

## Supporting Information

Appendix S1 **Search Strings and results.**
(DOCX)

Checklist S1 **PRISMA checklist.**
(DOC)
