# Correction: Psychological Group-Treatments of Social Anxiety Disorder: A Meta-Analysis

**DOI:** 10.1371/annotation/5f2f7ff4-ecfd-4a41-a162-34c1dd0c962a

**Published:** 2013-12-06

**Authors:** Hanna Wersebe, Marit Sijbrandij, Pim Cuijpers

This article has been republished to correct the following issue: the main figures of the paper were initially published as supporting information.

Please download the article again for the corrected version. 

