# Supplementary material for: Psychological Group-Treatments of Social Anxiety Disorder: A Meta-Analysis
Source: PLoS One. 2013 Nov 15;8(11):e79034. doi: 10.1371/journal.pone.0079034 (PMC3829844; doi:10.1371/journal.pone.0079034)
Supplement: Appendix S1 — Search Strings and results. (DOCX) [file pone.0079034.s001.docx]

**Apprendix S1**

**A. Search strings and results**

| **DATABASE** | **SEARCH STRING** | **Hits** | **DATE** |
| --- | --- | --- | --- |
| PUBMED | "social phobia"[All Fields] OR "social phobic"[All Fields] OR "social anxiety"[All Fields] OR "public-speaking anxiety"[All Fields] OR "public-speaking fear"[All Fields] OR "speaking anxiety"[All Fields] OR "speaking fear"[All Fields] AND ("humans"[MeSH Terms] AND Randomized Controlled Trial[ptyp] AND "adult"[MeSH Terms]) | 296 | 22 March 2012 |
| COCHRANE | “social phobia” or “social phobic” or “social anxiety” or “public-speaking anxiety” or “public-speaking fear” or “speaking anxiety” or “speaking fear” | 752 | 22 March 2012 |
| PSYCINFO | “social phobia” or “social phobic” or “social anxiety” or “public-speaking anxiety” or “public-speaking fear” or “speaking anxiety” or “speaking fear”  Limiters - Age Groups: Adulthood (18 yrs & older); Population Group: Human; Methodology: TREATMENT OUTCOME/CLINICAL TRIAL | 246 | 22 March 2012 |
| EMBASE | 'social phobia'/exp OR 'social phobia' OR 'social phobic' OR 'social anxiety' OR 'public-speaking anxiety' OR 'public-speaking fear' OR 'speaking anxiety' OR 'speaking fear'  AND 'clinical trial'/de AND ('article'/it OR 'conference abstract'/it OR 'conference paper'/it) | 661 | 22 March 2012 |
| **TOTAL** |  | **1955** |  |
|  | **ATER REMOVAL OF DUPLICATES** | **1228** |  |

**Excluded based on title and abstract: 976**

**Total retrieved pdf’s: 252**
